# *Trichinella spiralis* Paramyosin Alleviates Collagen-Induced Arthritis in Mice by Modulating CD4^+^ T Cell Differentiation

**DOI:** 10.3390/ijms25126706

**Published:** 2024-06-18

**Authors:** Dongwan Zhang, Wang Jiang, Yan Yu, Jingjing Huang, Zhihui Jia, Yuli Cheng, Xinping Zhu

**Affiliations:** Department of Medical Microbiology and Parasitology, School of Basic Medical Sciences, Capital Medical University, Beijing 100069, China; dongwanzhang08@outlook.com (D.Z.); jiangwang@mail.ccmu.edu.cn (W.J.); yuyan9612@mail.ccmu.edu.cn (Y.Y.); huangjj@ccmu.edu.cn (J.H.); jiazhihui639@163.com (Z.J.)

**Keywords:** *Trichinella spiralis* paramyosin, CD4^+^ T cells, T cell differentiation, collagen-induced arthritis, hygiene hypothesis

## Abstract

Rheumatoid arthritis (RA) is an autoimmune disease that significantly impacts quality of life by disrupting CD4^+^ T cell immune homeostasis. The identification of a low-side-effect drug for RA treatment is urgently needed. Our previous study suggests that *Trichinella spiralis* paramyosin (*Ts*-Pmy) has immunomodulatory effects, but its potential effect on CD4^+^ T cell response in RA remains unclear. In this study, we used a murine model to investigate the role of r*Ts*-Pmy in regulating CD4^+^ T cell differentiation in collagen-induced arthritis (CIA). Additionally, we assessed the impact of r*Ts*-Pmy on CD4^+^ T cell differentiation towards the Th1 and Th17 phenotypes, which are associated with inflammatory responses in arthritis, using in vitro assays. The results demonstrated that r*Ts*-Pmy administration reduced arthritis severity by inhibiting Th1 and Th17 response while enhancing Treg response. Prophylactic administration of *Ts*-Pmy showed superior efficacy on CIA compared to therapeutic administration. Furthermore, in vitro assays demonstrated that r*Ts*-Pmy could inhibit the differentiation of CD4^+^ T cells into Th1 and Th17 while inducing the production of Tregs, suggesting a potential mechanism underlying its therapeutic effects. This study suggests that *Ts*-Pmy may ameliorate CIA by restoring the immune balance of CD4^+^ T cells and provides new insights into the mechanism through which helminth-derived proteins exert their effects on autoimmune diseases.

## 1. Introduction

Rheumatoid arthritis (RA) is an autoimmune disease characterized by systemic, symmetrical joint involvement, with features including infiltration of inflammatory cells into the joint space, bone destruction, erosion of cartilage, and formation of vascular proliferation [1,2,3]. The prevalence of RA is approximately 0.5–1% of the global population [4,5,6,7], with an annual incidence rate of around 3 cases per 1000 individuals [8]. RA can lead to physical deformities in affected limbs, as well as significant burdens, both physical and economic, substantially compromising the quality of life of patients. The current first-line treatments for RA in clinical practice encompass disease-modifying anti-rheumatic drugs, nonsteroidal anti-inflammatory drugs, and glucocorticoids. Emerging RA medications such as tumor necrosis factor (TNF) inhibitors demonstrate notable therapeutic efficacy. However, they carry an elevated risk of infections and severe side effects, including hepatic and neurological failure [9]. Thus, there is an urgent need to identify medications capable of alleviating RA symptoms with relatively fewer adverse effects, given the considerable number of RA patients.

CD4^+^ T cells are considered crucial in the pathogenesis of RA. Upon antigen presentation by antigen-presenting cells (APCs) such as dendritic cells, CD4^+^ T cells are activated abnormally, showing an imbalance of Th1/Th2 and Th17/Treg, and migrate to the synovium, where they induce the activation and differentiation of macrophages and B cells through the secretion of cytokines or cell–cell contact [10,11]. CD4^+^ T cells in the joints of RA patients can activate macrophages through cell-to-cell contact, leading to the production of pro-inflammatory cytokines such as TNF, IL-1, and IL-6, which promote osteoclast differentiation, ultimately resulting in bone and cartilage destruction [12]. The secretion of cytokines by CD4^+^ T cells stimulate B cells to differentiate into plasma cells, producing autoantibodies. Cytokines secreted by CD4^+^ T cells, such as IL-17A, can stimulate synovial fibroblasts to differentiate into an invasive phenotype, leading to synovial hyperplasia and the formation of pannus and vasculature [13].

Since the proposition of the hygiene hypothesis in 1989 [14], numerous studies have demonstrated a negative correlation between helminth infections and the incidence of autoimmune diseases such as type 1 diabetes, multiple sclerosis, and inflammatory bowel disease [15]. As research has progressed, it has been found that single components of helminth excretory–secretory proteins or helminth-derived proteins, such as the excretory–secretory antigen-62 (ES-62) from the *Acanthocheilonema viteae* [16], the cystatin protease inhibitor from the *Schistosoma japonicum* (*Sj*Cystatin) [17], and the anti-inflammatory protein-1 from *Necator americanus* (*Na*-AIP-1) [18], can ameliorate CIA. Previous studies in our laboratory have revealed that infection with *Trichinella spiralis* can mitigate RA by modulating the balance of CD4^+^ T cell differentiation [19]. RA patients exhibit significant infiltration of inflammatory cells in the synovium, among which Th1 and Th17 cells are pivotal in mediating autoimmune reactions [20,21]. Thus, the identification of a single protein in *Trichinella spiralis* that regulates the function of CD4^+^ T cells is possible.

In 2008, our laboratory first cloned and identified the *Trichinella spiralis* paramyosin (*Ts*-Pmy) from its cDNA library [22]. This protein consists of 885 amino acids and has a size of 102 kDa. It is present on the surface of *Trichinella spiralis* at different stages and has also been found in the excretory–secretory products of adults and muscle larvae. Subsequent studies have revealed that *Ts*-Pmy possesses immunomodulatory properties and can alleviate inflammatory bowel disease, an autoimmune disease, by regulating CD4^+^ T cell-mediated immune dysregulation [23]. However, whether *Ts*-Pmy can exert an attenuative effect in the progression of RA by modulating the differentiation of CD4^+^ T cells remain unclear.

This study investigates the alleviating effect of recombinant *Ts*-Pmy (r*Ts*-Pmy) on the disease progression of collagen-induced arthritis (CIA) in mice, aiming to identify a biologic agent with minimal side effects that effectively alleviates the activity of RA in patients.

## 2. Results

### 2.1. Administration of rTs-Pmy Mitigates Collagen-Induced Arthritis in Mice

**Figure 1:**
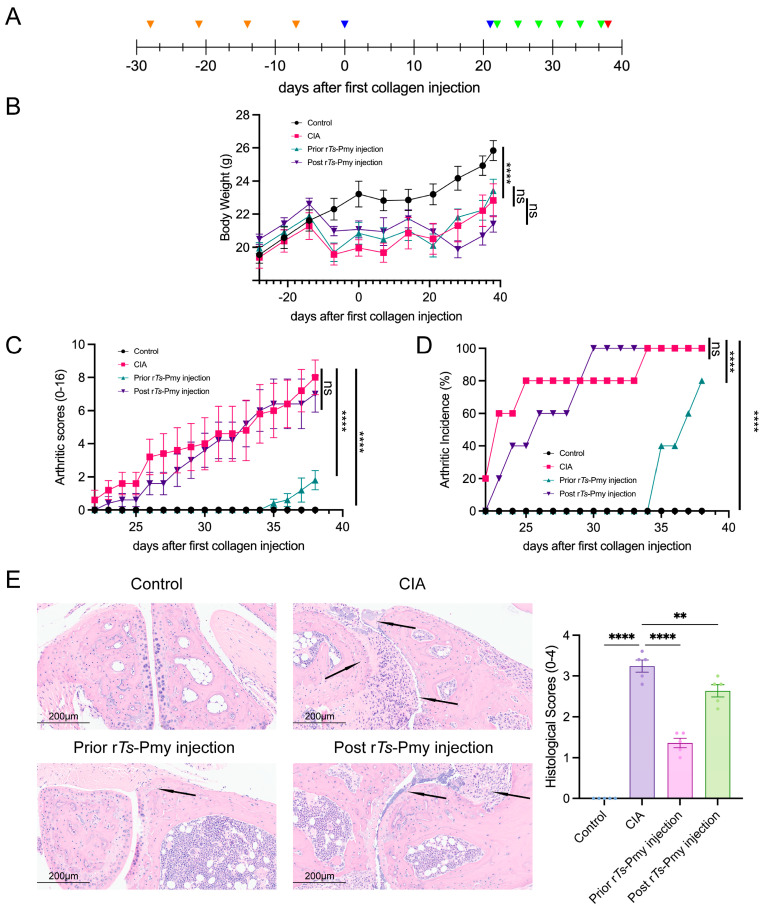
Mitigable effects of r*Ts*-Pmy on collagen-induced arthritis (CIA) in DBA/1 mice. (**A**) The regimen of study including the induction of CIA and treatment with r*Ts*-Pmy. Collagen-induced arthritis was induced in male DBA/1 mice. Yellow: prior r*Ts*-Pmy injection group, green: post r*Ts*-Pmy injection group, blue: CII immunization/boost, red: sacrifice. (**B**) Body weight; (**C**) arthritic scores (0–16); (**D**) arthritic Incidence (%); (**E**) hematoxylin and eosin staining of the representative inflamed joints in the hind paw of mice from different groups at day 38 after the first CII immunization, with the histological score shown on the right. Black arrows indicate synovial hyperplasia, cartilage destruction, inflammatory cell infiltration, and pannus formation. Scale bar: 200 µm. *n* = 5/group, data are shown with mean ± SEM (** *p* < 0.01 and **** *p* < 0.0001 vs. CIA group by one-way ANOVA test, ns indicates no significant difference).

To investigate the impact of r*Ts*-Pmy intervention on the progression of CIA, mice were randomly divided into four groups, a CIA model was established, and r*Ts*-Pmy injections were administered (Figure 1A). The body weight in the CIA group was significantly lower than that in the control group, while the weight was not significantly changed by prophylactic or therapeutic r*Ts*-Pmy intervention (Figure 1B). The arthritic score and arthritis incidence rate of the prior r*Ts*-Pmy injection group were significantly lower than those of the CIA group, with a delayed onset time observed for the prior injection group (Figure 1C,D). The examination of inflammatory cell infiltration in the paw joints was conducted following hematoxylin and eosin (HE) staining. Histological scoring was performed to assess the level of joint damage, utilizing predetermined criteria outlined in the Materials and Methods section (Table 1). Both prophylactic and therapeutic administration of r*Ts*-Pmy resulted in a significant reduction in paw joint damage in mice (Figure 1E). These findings suggest that the administration of r*Ts*-Pmy in CIA mice, particularly when administered prophylactically, can effectively mitigate disease severity and impede disease progression.

### 2.2. Administration of rTs-Pmy Alleviates the Imbalance of Systemic CD4^+^ T Cell Inflammation Caused by Arthritis, and Prophylactic Injection Generates Tregs

Blood can transport immune cells to all parts of the body, and the spleen, as the largest lymphoid organ in the body, is the place where mature T cells and B cells settle, and it can reflect the overall level of the body’s systemic immune response. To determine the impact of r*Ts*-Pmy on CD4^+^ T cell differentiation, we analyzed both serum and supernatants from splenocytes for CD4^+^ T cell-derived cytokines.

**Figure 2:**
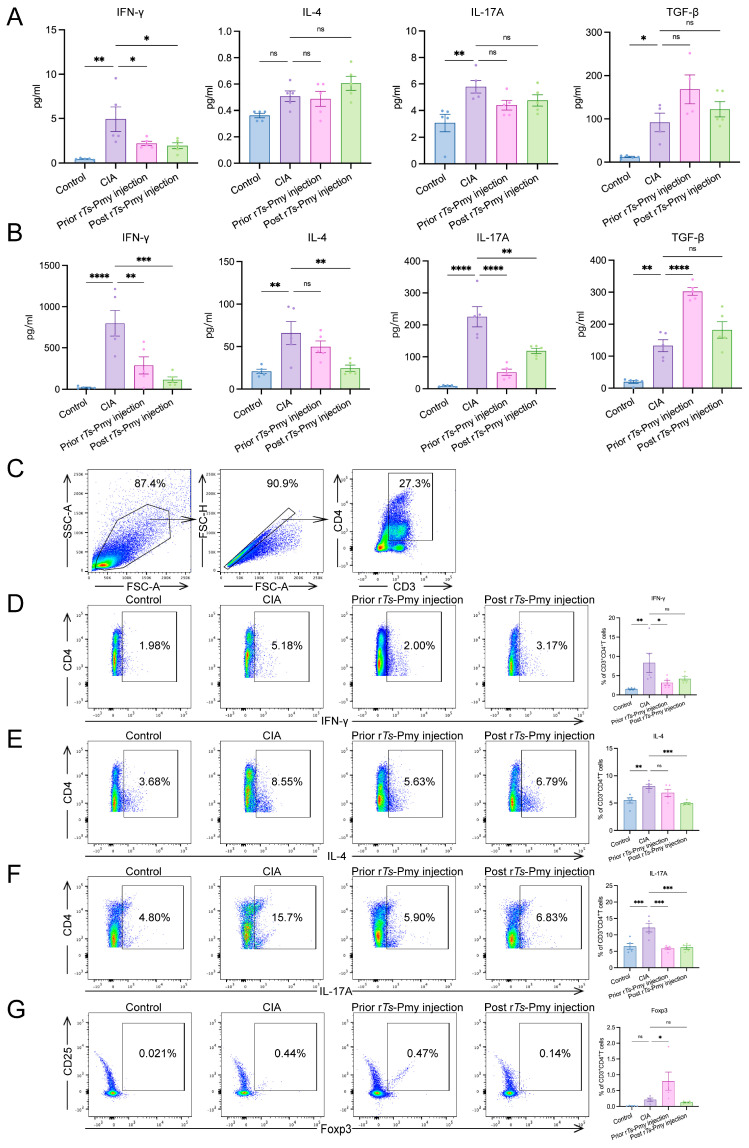
Administration of r*Ts*-Pmy alleviates systemic CD4^+^ T cell immune imbalance in CIA mice. (**A**) The concentration of serum cytokines in of mice different groups; (**B**) the concentration of cytokines in the supernatant of mouse splenocytes stimulated with Cell Stimulation Cocktail for 16 h; (**C**) flow cytometry gating strategy for CD3^+^CD4^+^ T cells. Single cells were gated from lymphocytes, and CD3^+^CD4^+^ T cells were gated from single cells; (**D**–**G**) the proportion of Th1 (**D**), Th2 (**E**), Th17 (**F**) cells and Tregs (**G**) in CD3^+^CD4^+^ T cells. *n* = 5 mice/group, data are shown with mean ± SEM (* *p* < 0.05, ** *p* < 0.01, *** *p* < 0.001, and **** *p* < 0.0001 vs. CIA group by one-way ANOVA test, ns indicates no significant difference).

As shown in Figure 2A, serum levels of IFN-γ, IL-17A, and TGF-β were markedly elevated in CIA group compared to the control group, while IL-4 exhibited a slight increase. The prophylactic or therapeutic administration of r*Ts*-Pmy significantly reduced IFN-γ levels in the serum of CIA mice, with a certain inhibitory effect on IL-17A. Additionally, both regimens resulted in an upward trend in TGF-β levels. However, no significant differences in IL-4 levels were observed among the groups. In the culture supernatant of splenocytes stimulated with a cocktail, levels of IFN-γ, IL-4, IL-17A, and TGF-β were significantly elevated in the CIA group compared to the control group. Compared with the CIA group, the secretion levels of IFN-γ and IL-17A in the prior r*Ts*-Pmy injection group were significantly decreased, and the secretion levels of TGF-β were considerably increased. In the post r*Ts*-Pmy injection group, a similar trend was observed. Compared to the CIA group, the post r*Ts*-Pmy injection group exhibited significantly decreased secretion levels of IFN-γ, IL-4, and IL-17A, while the secretion level of TGF-β showed an upward trend (Figure 2B).

To further investigate the role of CD4^+^ T cell subsets in the progression of CIA and the impact of r*Ts*-Pmy on immune balance in CIA mice, flow cytometry was employed to identify these cell populations in mouse splenocytes. Compared to the control group, a notable increase in the proportion of Th1 (CD4^+^IFN-γ^+^), Th2 (CD4^+^IL-4^+^), and Th17 (CD4^+^IL-17A^+^) cells was observed in the CIA group. In contrast, the prior r*Ts*-Pmy injection group exhibited a significant reduction in the proportion of Th1 and Th17 cells within the CD3^+^CD4^+^ T cell population compared to the CIA group. However, the proportion of Th2 cells in the prior r*Ts*-Pmy injection group did not exhibit a significant difference compared to the CIA group, while the proportion of Treg (CD4^+^CD25^+^Foxp3^+^) cells showed a significant increase. In the post r*Ts*-Pmy injection group, a slightly downward trend was found in the proportion of Th1 cells compared to the CIA group. However, the proportion of Th2 cells was significantly lower. Notably, the proportion of Th17 cells was significantly lower compared to the CIA group, while there was no significant difference observed in Treg cells (Figure 2C–G).

These results demonstrate that the administration of r*Ts*-Pmy in mice can alleviate the systemic imbalance of CD4^+^ T cell subsets. Furthermore, prior administration of r*Ts*-Pmy exhibits a stronger inhibitory effect on inflammatory responses mediated by both Th1 and Th17 subsets while promoting the generation of the Treg population in vivo.

### 2.3. Administration of rTs-Pmy Alleviates the Degree of Paw Joint Injury in CIA Mice by Regulating CD4^+^ T Cell Imbalance

**Figure 3:**
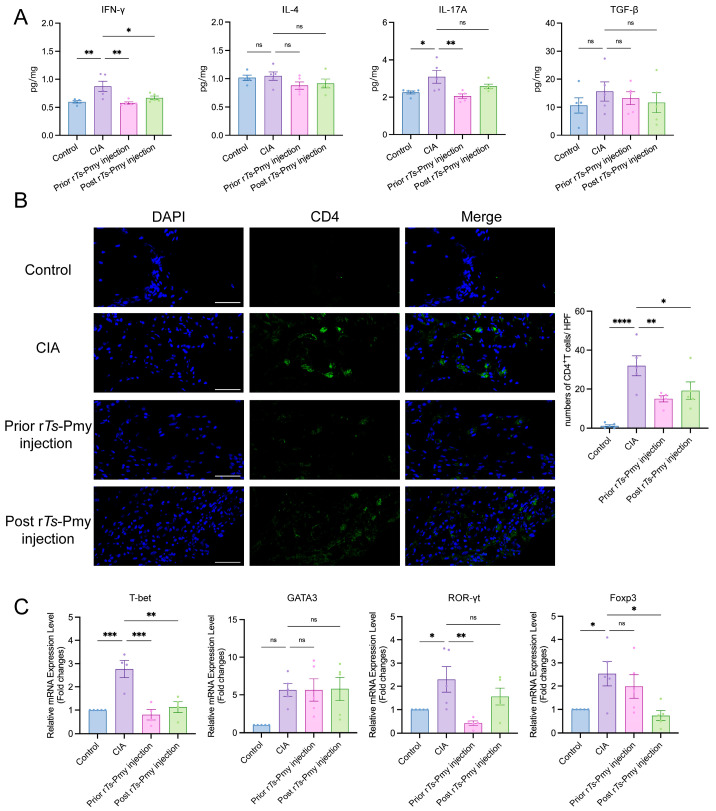
Administration of r*Ts*-Pmy ameliorated the immune imbalance of CD4^+^ T cells in CIA mice paw joints. (**A**) The concentration of cytokines in paws of mice in each group; (**B**) number of CD4^+^ T cells in paws, scale bar: 50 μm; (**C**) the expression level of transcription factors in CD4^+^ T cells. *n* = 5/group, data are shown with mean ± SEM (* *p* < 0.05, ** *p* < 0.01, *** *p* < 0.001, and **** *p* < 0.0001 vs. CIA group by one-way ANOVA test, ns indicates no significant difference).

To investigate the local inflammation at the affected site in CIA mice after the administration of r*Ts*-Pmy, the expression levels of IFN-γ, IL-4, and IL-17A in the paw homogenates were assessed using Luminex, while the expression level of TGF-β was detected by ELISA. The results revealed that the concentrations of IFN-γ and IL-17A in the CIA group were significantly higher than those in the control group. However, these two indicators were effectively reduced in the prior r*Ts*-Pmy injection group compared to the CIA group. In the post r*Ts*-Pmy injection group, there was a substantial decrease in IFN-γ, while a slight decrease was observed in IL-17A (Figure 3A).

To investigate the impact of r*Ts*-Pmy on CD4^+^ T cells in the joint cavity of mice, the infiltration of CD4^+^ T cells in the hind paws was examined. The results showed that there was no obvious cell infiltration, while the joint cavity structure of the paw of the CIA group was chaotic, and a large number of CD4^+^ T cells were infiltrated in the view field. The degree of infiltration was significantly higher than that of the control group, and compared with the CIA group, the joint structure of the prior r*Ts*-Pmy injection group was more intact, and the severity of CD4^+^ T cell infiltration was significantly reduced. Similar but less significant results were also found in post r*Ts*-Pmy injection group (Figure 3B).

To investigate the localized clustering of CD4^+^ T cells in the paw of mice within each group, the mRNA expression levels of *T-bet*, *GATA3*, retinoic orphan receptor γt (*ROR-γt*), and forkhead box P3 (*Foxp3*) corresponding to Th1, Th2, Th17, and Treg cells were detected using RTq-PCR. As shown in Figure 3C, the mRNA expression level of *T-bet* was notably reduced in both the prior r*Ts*-Pmy injection group and post r*Ts*-Pmy injection group compared to the CIA group. Notably, the expression level of *ROR-γt* was markedly lower in the prior r*Ts*-Pmy injection group compared to the CIA group. In the post r*Ts*-Pmy injection group, *ROR-γt* expression exhibited a downward trend compared to the CIA group, while the *Foxp3* expression level was significantly diminished.

These results suggested that prophylactic administration of r*Ts*-Pmy can effectively improve the infiltration of CD4^+^ T cells in the paws of CIA mice and regulate the immune imbalance of CD4^+^ T cells.

### 2.4. rTs-Pmy Inhibits the Differentiation of CD4^+^ T Cells in the Direction of Th1 and Th17 through the Presentation of Bone-Marrow-Derived Dendritic Cells (BMDCs) In Vitro

**Figure 4:**
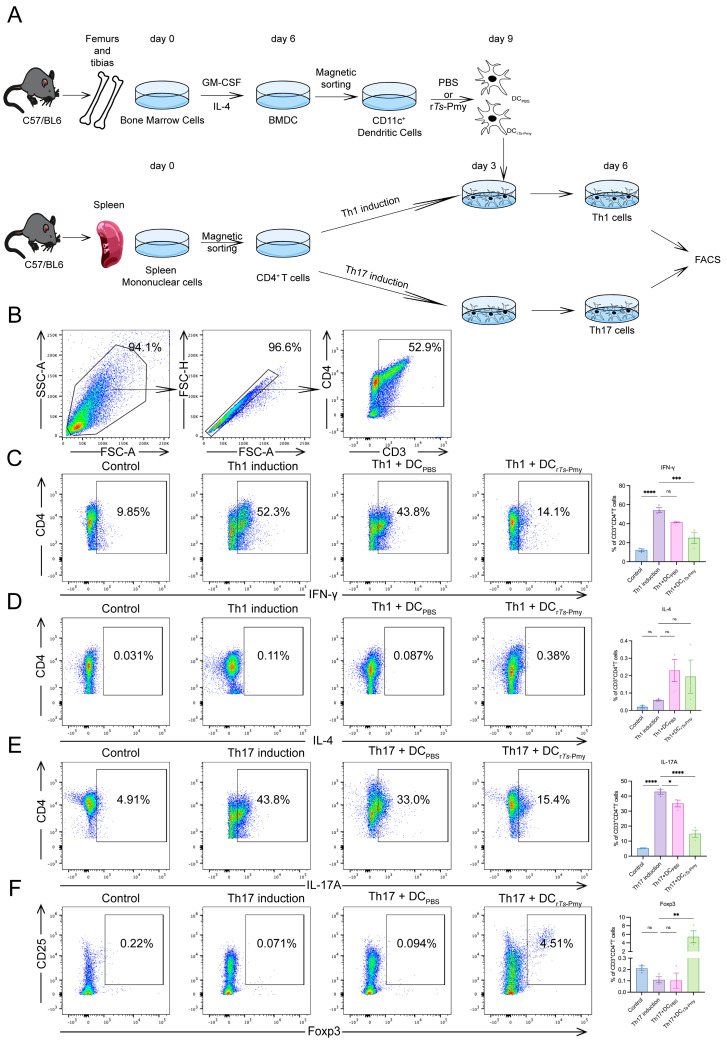
r*Ts*-Pmy inhibited the differentiation of CD4^+^ T cells in the direction of Th1 and Th17 in vitro through the presentation of BMDCs. (**A**) BMDC and Th1/Th17 co-culture experiment procedure; (**B**) gating strategy. Single cells were gated from lymphocytes, and CD3^+^CD4^+^ T cells were gated from single cells; (**C**) the proportion of CD4^+^IFN-γ^+^ Th1 cells in Th1 induction group; (**D**) the proportion of CD4^+^IL-4^+^ Th2 cells in Th1 induction group; (**E**) the proportion of CD4^+^IL-17A^+^ Th17 cells in Th17 induction group; (**F**) the proportion of CD25^+^Foxp3^+^ Th1 cells in Th17 induction group. *n* = 3/group, data are shown with mean ± SEM (* *p* < 0.05, ** *p* < 0.01, *** *p* < 0.001, and **** *p* < 0.0001 vs. Th1/17 induction group by one-way ANOVA test, ns indicates no significant difference).

To investigate whether r*Ts*-Pmy can inhibit the differentiation of CD4^+^ T cells into Th1 and Th17 subsets via dendritic cells (DCs), we co-incubated the r*Ts*-Pmy-treated DCs with CD4^+^ T cells in vitro following previously published protocols [24,25]. During this process, DCs pre-incubated with r*Ts*-Pmy (DC_r*Ts*-Pmy_) were added for intervention, while DCs cultured with an equal volume of PBS(DC_PBS_) served as a control. The proportions of Th cells and Treg cells were then assessed using flow cytometry. The experimental procedure is presented in Figure 4A.

The results of the Th1 induction group showed that CD4^+^ T cells differentiated into Th1 (over 50%) in the Th1 induction medium. Co-cultivation with DC_PBS_ did not significantly alter the proportion of Th1 cell differentiation. However, co-cultivation with DC_r*Ts*-Pmy_ resulted in a significant decrease in the proportion of Th1 cells in the induction system (Figure 4C). However, no significant difference was observed in the proportions of Th2 cells among the groups (Figure 4D).

The results from the Th17 induction group revealed that CD4^+^ T cells differentiated into Th17 (over 40%) in the Th17 induction medium. Co-cultivation with DC_PBS_ slightly decreased the proportion of Th17 cell differentiation. Conversely, co-cultivation with DC_r*Ts*-Pmy_ significantly reduced the proportion of Th17 cells in the induction system (Figure 4E). Treg cells significantly increased in the DC_r*Ts*-Pmy_ group, while no significant differences were observed in the Th17+DC_PBS_ group (Figure 4F).

These results suggest that r*Ts*-Pmy can inhibit the differentiation of CD4^+^ T cells in the direction of Th1 or Th17 while enhancing the direction of Treg through the presentation of DCs.

## 3. Discussion

Rheumatoid arthritis (RA), as an autoimmune disease, can lead to the symmetric destruction of bones and cartilage in small joints throughout the body, resulting in joint pain, limited mobility, and, in severe cases, significant limb deformities. The etiology of RA remains incompletely understood, with mainstream theories suggesting a multi-factorial causation involving genetic, environmental, and microbial factors [26,27,28]. Numerous studies have shown that helminth infection or intervention with helminth-derived proteins can alleviate the occurrence and progression of CIA in mice [29,30,31,32,33]. In our experiment, we observed that administration of r*Ts*-Pmy to mice could alleviate the severity of CIA, reduce the incidence rate, delay the onset time, and decrease the degree of inflammatory cell infiltration in the joints. Moreover, the preventive effect of r*Ts*-Pmy administration was superior to therapeutic treatment.

The dysregulation of the balance of Th1/Th2 and Th17/-Treg cells in RA patients is considered a key factor in the pathogenesis of RA. Our previous studies [34] have indicated that r*Ts*-Pmy can induce the generation of regulatory T cells through presentation by BMDCs, and its structure has been found to contain CD4^+^ T cell epitopes [35], further elucidating r*Ts*-Pmy as a unique protein of *Trichinella spiralis* possessing immunomodulatory properties and closely associated with CD4^+^ T cells. Recent investigations have revealed that *SJ*MHE1, derived from the *Schistosoma japonicum* heat shock protein 60, exerts immunomodulatory effects by reducing the secretion of Th1 and Th17 pro-inflammatory cytokines in CIA mice, while simultaneously increasing Treg cell populations, leading to significant alleviation of arthritis in the mice [36]. Additionally, the excretory–secretory antigen ES-62 from *Acanthocheilonema viteae* can downregulate myeloid differentiation factor 88, inhibiting the differentiation of Th17 cells and effectively alleviating the condition of CIA mice [37]. From our results, it is evident that prior administration of r*Ts*-Pmy can ameliorate the imbalance of CD4^+^ T cells in CIA mice, reduce systemic and local Th1- and Th17-type immune responses, and potentially induce Tregs, which is similar to the efficacy of other helminth-derived proteins such as *Schistosomiasis japonicum* cystatin and *Toxoplasma gondii* antigen lysate in alleviating CIA [38]. Many studies have shown that CD4^+^CD25^+^Foxp3^+^ Tregs have the ability to ameliorate the imbalance of Th17 inflammatory response in many autoimmune diseases [39,40,41]. A lack of CD4^+^CD25^+^Foxp3^+^ Tregs can cause autoimmune disease in both humans and mice [42,43]. In this study, CD4^+^CD25^+^Foxp3^+^ Tregs were successfully generated by r*Ts*-Pmy both in vivo and in vitro, and we believe that this is the key reason why r*Ts*-Pmy has the ability to alleviate collagen-induced arthritis in mice.

Interestingly, our results demonstrate a subtle difference in the trend of the representative cytokine IL-4, associated with type 2 immune response, among the serum and spleen samples from immunized mice and the affected paw region of diseased mice. One possible explanation is that r*Ts*-Pmy may not regulate the imbalance of CD4^+^ T cells in CIA mice primarily by inducing Th2-type immune responses. Another study suggested that *Trichinella spiralis* infection in treating CIA mice does not necessarily require the involvement of Th2-type cytokines such as IL-4, and even in the absence of STAT-6, the upstream transcription factor of Th2 cells, its regulatory effects can still be observed [44]. This finding provides a plausible explanation for the lack of a pronounced Th2-type immune response induced by r*Ts*-Pmy observed in this experiment. Another possible explanation could involve r*Ts*-Pmy triggering other immune cells to elicit a regulatory immune response. Another study found that *Anisakis simplex* r*As*-migration inhibitory factor can treat DSS-induced colitis by inducing IL-10 secretion and Treg production [45]. The differences in the sites of action of these different helminth-derived proteins and the different mechanisms of their action can be the next research direction. Due to the different research focuses, our research did not detect the changes in other cells, which is one of the possible directions for subsequent experiments.

Our experiment further confirmed that r*Ts*-Pmy could inhibit the differentiation of CD4^+^ T cells in the direction of Th1 and Th17 through the presentation of DCs, significantly increasing the proportion of Treg in the Th17 induction system. To observe if DC_r*Ts*-Pmy_ can intervene in the Th1 differentiation process, we added enough IL-4 antibody to the Th1 induction system to exclude potential interference. In the Th17 induction group, we observed a significant increase in CD4^+^CD25^+^Foxp3^+^ Tregs stimulated by r*Ts*-Pmy after presentation by DCs. We speculate that this increase in Tregs led to the suppression of Th17 cell differentiation and development within the system. Consequently, we postulate that r*Ts*-Pmy is a protein with immunomodulatory functions, capable of regulating the immune imbalance of CD4^+^ T cells.

In summary, our study analyzed the role of r*Ts*-Pmy as a novel immunomodulatory molecule in the immune balance of CD4^+^ T cells, which provided a research idea for the prevention and treatment of autoimmune diseases by helminth-derived molecules.

## 4. Materials and Methods

### 4.1. Animals

Male DBA/1 mice aged 6–8 weeks and male C57BL/6J mice aged 6–8 weeks were purchased from the Capital Medical University Laboratory Animal Services Center (Beijing, China). All animals were kept in a pathogen-free environment. All animal experimental procedures were approved by the Animal Care and Use Committee of Capital Medical University (approval number: AEEI-2015-160) and complied with the National Institutes of Health Guidelines for the Care and Use of Experimental Animals.

### 4.2. Expression and Purification of Recombinant Trichinella Spiralis Paramyosin (rTs-Pmy)

Recombinant *Ts*-Pmy was expressed and purified by an insect cell/baculovirus protein expression system following a previous description [23]. Briefly, the Bac-to-Bac baculovirus system was utilized to construct a recombinant baculovirus containing the *Ts*-Pmy gene sequence. Firstly, the pFastBac *E. coli* vector was combined with the baculovirus using site-specific Tn7 transposon, resulting in the first generation of the recombinant baculovirus (P1). This recombinant virus was then amplified in sf9 insect cells cultured in adherent conditions for two generations (P3), followed by transfer of the P3 virus to sf9 cells cultured in suspension for protein expression. After 3 days, insect cells were collected and lysed using a high-pressure homogenizer. The resulting lysate, containing r*Ts*-Pmy inclusion bodies (IBs), was obtained after centrifugation. Subsequently, the IBs were dissolved overnight in 6 mol/L guanidine hydrochloride (IB solution buffer) and then subjected to purification using nickel affinity chromatography. Impurities were removed by wash buffer (IB solution buffer containing 10 mmol/L or 50 mmol/L imidazole), and pure r*Ts*-Pmy with a His tag was eluted using elution buffer (IB solution buffer containing 500 mmol/L imidazole). The eluted, denatured r*Ts*-Pmy was dialyzed in 20 mmol/L Tris-HCl (pH 7.9) using the Novagen protein refolding kit to refold the denatured protein. The refolded r*Ts*-Pmy was further subjected to three rounds of gradient dialysis for 3 h each, using 20 mmol/L Tris-HCl (pH 7.9) dialysis buffer, followed by 3 h of PBS dialysis to dissolve the refolded r*Ts*-Pmy in PBS. The resulting solution was characterized by SDS-PAGE and was recognized by anti-*Ts*-Pmy monoclonal antibody 9G3 that specifically recognized *Ts*-Pmy which was previously produced [46]. The purified r*Ts*-Pmy was filtered through a 0.22 μm membrane for sterilization and stored at −80 °C for later use.

### 4.3. Construction of CIA Mice Model and Administration of rTs-Pmy

Twenty male DBA/1 mice, aged 7–10 weeks, were randomly divided into four groups, with 5 mice in each: control group, CIA group, prior r*Ts*-Pmy injection group, and post r*Ts*-Pmy injection group. The control group received no treatment, while the mice in the other three groups were subjected to the construction of the collagen-induced arthritis (CIA) model according to the methods previously described [19]. On the day of the first bovine type II collagen (CII, Chondrex, Woodinville, WA, USA) immunization (day 0), the CIA group, prior r*Ts*-Pmy injection group, and post r*Ts*-Pmy injection group were immunized by injecting 100 μL per mouse of a mixture of CII and complete Freund’s adjuvant (CFA, Chondrex, USA) intradermally into the base of the mouse’s tail, following emulsification on ice until no spreading occurred on the water surface. On day 21, the mice were re-immunized by injecting 100 μL per mouse of a mixture of CII and incomplete Freund’s adjuvant (IFA, Chondrex, Woodinville, WA, USA) intradermally at multiple sites on the back, after complete emulsification, to establish the CIA model. In the prior r*Ts*-Pmy injection group, r*Ts*-Pmy was injected intraperitoneally at a dose of 20 μg per mouse on day -28, with subsequent injections every 7 days until the initial collagen immunization, totaling 4 injections. In the post r*Ts*-Pmy injection group, r*Ts*-Pmy was injected intraperitoneally after the onset of disease symptoms (day 21), at the same dose as the prior r*Ts*-Pmy injection group, with injections every 3 days, totaling 5 injections. All mice were sacrificed on day 38 (Figure 1A).

### 4.4. Body Weight, Arthritic Score, and Arthritis Incidence Recording

The body weight, arthritic score, and arthritis incidence rate were observed and recorded from day 22. The arthritic scoring standard is shown in Table 1. The arthritic score of each mouse was the sum of the total scores (0–16).

### 4.5. Hematoxylin–Eosin (H&E) Staining and Histological Scoring

The fresh hind paws were collected and fixed in 4% paraformaldehyde. After decalcification, the tissues were dehydrated with alcohol, embedded in paraffin, and sectioned. The sectioned paw was attached to a glass slide and deparaffinized with xylene. The sections were then stained with H&E after rehydration. The histological score was evaluated as described in Table 2.

### 4.6. Spleen Mononuclear Cell Isolation and Culturing

On day 38, spleens were aseptically collected from mice. Mononuclear cells from the spleen were isolated according to the instructions of the mouse lymphocyte separation solution (Dakewei, Shanghai, China). After cell counting, 5 × 10^5^ cells per well were seeded into 24-well plates. The culture medium consisted of RPMI 1640 complete medium containing 1% penicillin–streptomycin (Solarbio, Beijing, China) and 10% fetal bovine serum (FBS, Biological Industries, Cromwell, CT, USA).

For cytokine detection, the culture medium was additionally supplemented with 1× Cell Stimulation Cocktail (without protein transport inhibitors) (TONBO, San Diego, CA, USA), while for cell differentiation detection, the culture medium was supplemented with 1× Cell Stimulation Cocktail (TONBO, USA). The cells were cultured for 16 h.

### 4.7. Luminex

Extraction of mice paw full protein was as follows: Hind paws were ground in PBS containing 1×protease inhibitor cocktail (MCE, Monmouth Junction, NJ, USA) and PMSF (Yeasen, Shanghai, China) at 4 °C, and the supernatant was obtained after centrifugation. Supernatants from the paw were collected and determined using Luminex bead array technology. Cytokine levels were measured by Luminex multianalyte technology, using the Procarta Plex™ Analyst 1.0 with magnetic beads, in accordance with the manufacturer’s protocols (affymetrix, eBioscience, San Diego, CA, USA).

### 4.8. Immunofluorescence Staining

Paw synovial tissues were incubated with a primary antibody (mice anti-mice CD4) (1:200, ABM40070, Abkine, St Wellesley, MA, USA). Goat anti-mouse IgG H&L (Alexa Fluor^®^ 488) (1:500, ab150113, Abcam, Cambridge, UK) was used as a secondary antibody Nuclei were stained with DAPI, and the images were observed under a fluorescence microscope (Nikon, Tokyo, Japan) and analyzed by K-Viewer System (Konfoong Biotech, Ningbo, China).

### 4.9. Enzyme-Linked Immunosorbent Assay

The cell culture supernatant was collected, and the secretion levels of interferon-γ (IFN-γ), interleukin-4 (IL-4), IL-17A, and transforming growth factor-β (TGF-β) were determined via ELISA assay kits (Thermo Scientific, Waltham, MA, USA) according to the instructions. The content of inflammatory cytokines in the samples was calculated by a standard curve.

### 4.10. Flow Cytometry

The cultured cells were collected and washed twice with PBS, followed by incubation with blocking antibody anti-CD16/32 (Clone: 2.4G2, TONBO, USA) at 4 °C in the dark. Surface staining was then performed by adding anti-CD3e-PerCP5.5 (Clone: 145-2C11, TONBO, USA), anti-CD4-APC-Cy7 (Clone: GK1.5, TONBO, USA), and anti-CD25-PE-CF594 (Clone: PC61, BioLegend, San Diego, CA, USA). After 30 min of incubation at 4 °C in the dark, cells were washed with PBS, followed by the addition of Transcription Factor Fix/Perm working solution (TONBO, USA) and incubation at 4 °C for 1 h. After the Transcription Factor Perm working solution (TONBO, USA) was washed off, the cells were stained with intracellular/nuclear antibodies, namely anti-IFN-γ-violetFluor450 (Clone: XMG1.2, TONBO, USA), anti-IL-4-PE (Clone: 11B11, TONBO, USA), anti-IL-17A-PE-Cy7 (eBio17B7, eBioscience, USA), and anti-Foxp3-APC (Clone: 3G3, TONBO, USA), and incubated at 4 °C in the dark for 30 min. Following staining, cells were washed once with Transcription Factor Perm working solution and once with PBS before analysis using a BD FACS Symphony flow cytometer. Data analysis was performed using FlowJo 10.8.1 (Becton Dickinson & Company, Milpitas, CA, USA).

### 4.11. RNA Extraction and Real-Time Quantitative PCR (RTq-PCR)

Paws’ skin was stripped and minced, and total RNA was extracted using Trizol regent and reverse-transcribed with HiScript III RT SuperMix for qPCR (+gDNA wiper) (Vazyme, Nanjing, China). RTq-PCR was performed using AceQ Universal SYBR qPCR Master Mix (Vazyme, China) with specific primers (Table 3).

### 4.12. Acquisition of Purified Bone-Marrow-Derived Dendritic Cells (BMDCs)

C57BL/6 mice aged 6–8 weeks were used to obtain bone-marrow-derived dendritic cells, modified based on the Inaba method [47]. Briefly, bone marrow was extracted from the femurs and tibias of mice by flushing; after passing through a 200-mesh sieve, red blood cells in the bone marrow were lysed using red blood cell lysis buffer (Solarbio, China). The harvested cells were cultured with RPMI 1640 supplemented with 10% FBS, 1% SP, 20 ng/mL IL-4 (Peprotech, Rocky Hill, NJ, USA), and 10 ng/mL GM-CSF (Peprotech, USA) in a Petri dish not treated with TC. On day 3, all the medium was carefully aspirated, PBS was added to the culture medium, and suspended cells were washed away. The medium was replenished with a medium containing the same components as the initial culture was replenished. On day 6, all cells were collected by pipetting. Then, they were washed and incubated with anti-mouse CD11c-coated magnetic beads for 10 min. After washing, CD11c^+^ DCs were purified by positive selection using MACS separation columns (Miltenyi Biotec, Bergisch, Germany) according to the manufacturer’s instructions.

### 4.13. Stimulation of DCs with rTs-Pmy In Vitro

Purified DCs (1 × 10^5^ cells/well) were cultured with 10 μg/mL r*Ts*-Pmy or PBS in the same volume as DC_r*Ts*-Pmy_ and DC_PBS_ in 6-well tissue culture plates in RPMI 1640 complete medium for 72 h.

### 4.14. Acquisition of Purified CD4^+^ T Cells and Th1/17 Induction

Naïve CD4^+^ T cells were isolated from C57BL/6 mice spleens using CD4^+^ T cell isolation kits (Miltenyi Biotec, Germany). Purified CD4^+^ T cells (1 × 10^6^) were incubated in a 24-well plate coated with 5 μg/mL anti-mouse-CD3 and anti-mouse-CD28 (BioLegend, USA).

To induce Th1 cell differentiation, CD4^+^ T cells were incubated in a Th1 induction medium containing recombinant mouse (rm)-IL-2 (10 ng/μL), rm-IL-12 (10 ng/μL), and anti-mouse IL-4 (10 μg/mL).

To induce Th17 cell differentiation, CD4^+^ T cells were incubated in Th17 induction medium containing rm-IL-6 (50 ng/mL), rm-IL-23 (5 ng/mL), recombinant human (rh)-TGF-β1 (10 ng/mL), anti-mouse IFN-γ (10 μg/mL), and anti-mouse IL-4 (10 μg/mL) (Peprotech, USA).

### 4.15. DC and CD4^+^ T Cell Co-Culture

The DC_PBS_ and DC_r*Ts*-Pmy_ (1 × 10^5^) were washed and then added to the Th1 and Th17 induction system (1 × 10^6^) on the third day after Th induction. Three days after co-culturing, the cells were harvested and stained for flow cytometry assay.

### 4.16. Statistical Analysis

GraphPad Prism version 9.0 (Graphpad Software) was used for statistical analysis. Data were displayed as mean ± SEM. All error bars represent SEM. One-way ANOVA with Tukey post hoc test was performed for comparison between groups. *p* values less than 0.05 were considered as indicative of significant differences (* *p* < 0.05, ** *p* < 0.01, *** *p* < 0.001, **** *p* < 0.0001).

## Figures and Tables

**Table 1:** Arthritic scoring standard.

Arthritic Score	Severity
0	healthy paws without swelling
1	1 swelling toe
2	2 swelling toes or swelling palm
3	1–2 swelling toes and swelling palm
4	3–4 swelling toes and swelling palm

**Table 2:** Histological Scoring standard.

Histological Score	Severity
0	healthy joint
1	less inflammatory cell infiltration
2	more inflammatory cell infiltration
3	more inflammatory cell infiltrates with narrow joint space and mild synovial hyperplasia
4	massive inflammatory cell infiltration with articular space stenosis, severe synovial hyperplasia, and structural changes in cartilage and bone

**Table 3:** Primers of real-time quantitative PCR.

Gene	Forward Sequence (5′ to 3′)	Reverse Sequence (5′ to 3′)
*T-bet*	GCCAGGGAACCGCTTATATG	GACGATCATCTGGGTCACATTGT
*GATA3*	ACGATCCAGCACAGGCAG	AGGATGTCCCTGCTCTCCTT
*ROR-γt*	ACAAATTGAAGTGATCCCTTGC	GGAGTAGGCCACATTACACTG
*Foxp3*	CCCAGGAAAGACAGCAACCTT	TTCTCACAACCAGGCCACTTG
*β-actin*	GAGAGGGAAATCGTGCGTGACA	ACCCAAGAAGGAAGGCTGGAAA

## Data Availability

Data is contained within the article.
